# Künstliche Intelligenz und berufliche Teilhabe in Deutschland: Ein narratives Review

**DOI:** 10.1007/s00103-026-04271-1

**Published:** 2026-07-22

**Authors:** Mareike Decker, Sara Hamideh Kerdar, Patricia Traub

**Affiliations:** 1 REHADAT, Institut der deutschen Wirtschaft Köln e.V. (IW), Konrad-Adenauer-Ufer 21, 50668 Köln, Deutschland; 2 Bundesanstalt für Arbeitsschutz und Arbeitsmedizin (BAuA), Friedrich-Henkel-Weg 1-25, 44149 Dortmund, Deutschland

**Keywords:** Künstliche Intelligenz, Berufliche Teilhabe, Menschen mit Behinderungen, Inklusion, Arbeit, Artificial intelligence, Occupational participation, People with disabilities, Inclusion, Work

## Abstract

Die Literatur zeigt, dass künstliche Intelligenz (KI) trotz bestehender Einschränkungen neue Möglichkeiten bieten kann, Menschen mit Behinderungen im Arbeitsleben zu unterstützen. Der aktuelle Forschungsstand in Deutschland zum Einsatz von KI zur Förderung beruflicher Teilhabe ist jedoch unübersichtlich. Ziel dieses narrativen Reviews ist es daher, einen aktuellen Überblick über Projekte und Publikationen zu geben, die in Deutschland durchgeführt beziehungsweise veröffentlicht wurden. Für den Zeitraum von 2020 bis 2025 wurde eine strukturierte Recherche in mehreren Datenbanken und auf einschlägigen Websites durchgeführt. Eingeschlossen wurden Projekte und Publikationen, die sich mit dem Einsatz von KI im beruflichen Kontext für Menschen mit Behinderungen befassen. In einer thematischen Analyse wurden Chancen und Herausforderungen des KI-Einsatzes herausgearbeitet. Es wurden 16 Projekte und 8 Publikationen identifiziert. Die Analyse zeigt, dass KI dazu beitragen kann, Barrieren zu überwinden, etwa durch die Unterstützung von Arbeitsprozessen und die Förderung von Selbstbestimmung. Zugleich werden Herausforderungen deutlich, insbesondere im Hinblick auf Datenschutz, mangelnde Transparenz bei der Datenverarbeitung sowie mögliche personelle und finanzielle Mehraufwände beim Einsatz KI-gestützter (assistiver) Technologien. Auf Grundlage der Analyse von Projekten und Publikationen schließt der Artikel mit praktischen Empfehlungen und Implikationen für die zukünftige Forschung zum Einsatz KI-gestützter Technologien zur Förderung beruflicher Teilhabe.

## Abstract (en)

The literature shows that, despite existing limitations, artificial intelligence (AI) can provide new opportunities to support people with disabilities at work. However, the current state of research in Germany on the use of AI to facilitate participation in working life is unclear. The aim of this narrative review is therefore to provide an up-to-date overview of projects and publications conducted or published in Germany. A structured search was carried out in several databases and relevant websites covering the period from 2020 to 2025. Projects and publications focusing on the use of AI in an occupational context for people with disabilities were included. A thematic analysis was conducted to identify the opportunities and challenges associated with the use of AI. A total of 16 projects and 8 publications were identified. The analysis shows that AI can help to overcome barriers, for example by supporting work processes and promoting self-determination. At the same time, challenges become apparent, particularly regarding data protection, a lack of transparency in data processing, and potential additional personnel and financial resources required when using AI-supported (assistive) technologies. Based on the analysis of projects and publications, the article concludes with practical recommendations and implications for future research on the implementation of AI-based technologies to promote participation in working life.

## Einleitung

In der wissenschaftlichen Literatur lässt sich in den letzten Jahren ein wachsendes Interesse an den Potenzialen, Auswirkungen und Grenzen künstlicher Intelligenz (KI) im Arbeitskontext beobachten. Ein zentraler Forschungsgegenstand ist beispielsweise die Zusammenarbeit zwischen Menschen und KI [1, 2]. Dabei wird untersucht, bei welchen Aufgaben KI nützlich sein kann (vgl. z. B. [3]), ob sie bestimmte Tätigkeiten ersetzen könnte [4] und inwiefern sie Kompetenzen von Beschäftigten fördern oder die Produktivität steigern kann [5].

Im Kontext von KI und beruflicher Teilhabe von Menschen mit Behinderungen wächst die Forschungsliteratur stetig. Studien zeigen, dass KI Teilhabechancen verbessern kann. Ein Beispiel ist die Integration von KI in bestehende Hilfsmittel, wie in Screenreader zur automatischen Bildbeschreibung. In einem Scoping-Review zu „KI-gestützten assistiven Technologien“ betont Alsaleh [6] die Bedeutung von KI unter anderem am Arbeitsplatz für Menschen mit Behinderungen, wo beispielsweise KI-gestützte robotische Arme Menschen mit körperlichen Behinderungen eine deutlich unabhängigere Ausführung von Arbeitsaufgaben ermöglichen. Außerdem können KI-gestützte Geräte Personen mit kognitiven Beeinträchtigungen durch adaptive Erinnerungen und strukturierte Planung im Zeitmanagement unterstützen [6].

Gleichzeitig legt die Forschung nahe, dass KI unbeabsichtigte negative Folgen unter anderem für Menschen mit Behinderungen haben kann. Beispielsweise wird darauf hingewiesen, dass bei der Nutzung von KI-gestützten Tools zur Zusammenstellung von Teams Menschen mit Behinderungen häufig nicht berücksichtigt werden [7]. In einem Scoping-Review identifizierten El Morr et al. [8] weitere Barrieren, darunter Datenschutz und ethische Probleme sowie die unzureichende Berücksichtigung der Belange von Menschen mit Behinderungen in der KI-Entwicklung.

In Deutschland lebten Ende 2023 knapp 7,9 Mio. Menschen mit einer anerkannten Schwerbehinderung (d. h. mit einem Grad der Behinderung von 50 oder mehr; [9]). Von ihnen sind knapp 3,1 Mio. im erwerbsfähigen Alter zwischen 15 und 65 Jahren [9]. Die Erwerbsquote der Menschen mit Behinderungen lag im Jahr 2023 bei 61,9 % und damit unter der von Menschen ohne Behinderungen (82,8 %; [10]). Trotz des Rechts auf Teilhabe an Arbeit und Beschäftigung nach Artikel 27 der Behindertenrechtskonvention der Vereinten Nationen (UN-BRK; [11]) und der Bedeutung von Arbeit für das Wohlbefinden und die Lebensqualität [12] fehlen bislang hinreichende Erkenntnisse zu den potenziellen Chancen und Herausforderungen von KI für die berufliche Teilhabe von Menschen mit Behinderungen in Deutschland.

Literaturrecherchen wie narrative Reviews bieten einen umfassenden Überblick über die Forschung zu einem Thema [13, 14]. Ziel dieses Beitrags ist, den Forschungsstand der Nutzung KI-gestützter Technologien für die berufliche Teilhabe von Menschen mit Behinderungen in Deutschland zu beschreiben. Zudem werden die mit KI-Technologien verbundenen Chancen und Herausforderungen identifiziert. Abschließend werden wissenschaftliche und praktische Implikationen abgeleitet.

## Methodik

In dieser Übersicht wurden ausschließlich Projekte, wissenschaftliche Studien und Projektberichte aus Deutschland berücksichtigt, da die Implementierung und Förderung KI-gestützter Assistenztechnologien maßgeblich durch nationale rechtliche und strukturelle Rahmenbedingungen bestimmt sind. Die Literaturrecherche erfolgte in mehreren wissenschaftlichen Datenbanken, darunter PsycInfo und Embase sowie in der Suchmaschine Google Scholar. Die Suchbegriffe bestanden aus einer Kombination verschiedener Begriffe auf Englisch und Deutsch zu den Themen künstliche Intelligenz, Arbeit und Behinderung. Sie wurden in Verbindung mit Trunkierungen durch Asterisk (*) angewendet, um Wortstammvariationen abzudecken, und mit booleschen Operatoren (AND und OR) logisch verknüpft. Ein Suchstring lautet z. B. („Künstliche Intelligenz“ OR KI) AND (Teilhabe OR Inklusion) AND (Arbeit* OR Beruf*) AND (Behinderung* OR Beeinträchtigung*). Ergänzend wurde eine manuelle Suche über Google, in einschlägigen Fachzeitschriften und auf Websites von Universitäten durchgeführt, insbesondere dann, wenn zu neueren oder laufenden Projekten noch keine Veröffentlichungen zum Recherchezeitpunkt vorlagen. In einem Fall wurden projektspezifische Informationen über das Netzwerk „digitale Assistenzsysteme am Arbeitsplatz (daaap-Netzwerk^1^)“ eingeholt.

Für eine umfassende Studienbasis wurden Veröffentlichungen und Projekte aus den Jahren 2020 bis 2025 einbezogen, in denen KI-Technologien für Menschen mit Behinderungen im Arbeitskontext und die potenziellen Auswirkungen untersucht wurden. Dazu zählen unter anderem Feldstudien, Pilotprojekte und Expert:innen-Workshops. Die Autor:innen der Publikationen oder die Ansprechpersonen der Projekte wurden im Zweifelsfall kontaktiert, beispielsweise um sicherzustellen, dass KI Gegenstand der Studie oder des Projekts war.

Ausgeschlossen wurden Arbeiten ohne eindeutig erkennbaren Einsatz von KI-Technologien oder ohne klaren Arbeitsbezug – etwa aufgrund einer Vermischung von Arbeits- und Alltagssituationen ohne eindeutige Trennung des beruflichen Settings. Ebenso ausgeschlossen wurden Studien mit Teilnehmenden außerhalb des Erwerbsalters (z. B. Kinder oder ältere Erwachsene) sowie ohne hinreichende Angaben zu den eingesetzten KI-Funktionen oder zu potenziellen Chancen und Herausforderungen für die Zielgruppe.

Da zu den identifizierten Projekten in der Regel nur begrenzte Informationen vorlagen, wurde die thematische Analyse ausschließlich für die Publikationen durchgeführt. Die Ergebnisse der ausgewerteten Publikationen wurden dokumentiert und nach den 6 Schritten der thematischen Analyse nach Braun und Clarke [15] Themenclustern (Haupt- und Subthemen) zugeordnet. Dabei wurden die folgenden Schritte durchgeführt: 1. Vertrautmachen mit den Daten, 2. Erstellen von ersten Codes, 3. Themensuche, 4. Überprüfen der Themen, 5. Definieren der Themen, 6. abschließende Analyse.

## Ergebnisse

Zunächst werden Übersichten zu den einbezogenen Projekten, Berichten und wissenschaftlichen Publikationen präsentiert, gefolgt von einer detaillierten Beschreibung der Ergebnisse aus der thematischen Analyse der Publikationen.

### Übersichten

Sechzehn einbezogene Projekte werden mit ihren Zielen und Inhalten in Tab. 1 aufgeführt, die Informationen zu den 8 inkludierten Publikationen in Tab. 2. Das Projekt KI.ASSIST^2^, ein größeres Projekt zum Thema KI und berufliche Teilhabe in Deutschland, umfasst ein breites Spektrum an Informationen, Arbeitspaketen und Methoden (Literaturrecherche, Workshops und Feldstudien in Lern- und Experimentierräumen). Daher wird KI.ASSIST als Projekt in Tab. 1 und die für diesen Beitrag relevanten projektbezogenen Publikationen in Tab. 2 dargestellt.

**Tab. 1:** Eingeschlossene Projekte zu KI-Technologien im Arbeitskontext für Menschen mit Behinderungen im Zeitraum 2020–2025 in Deutschland

Projekttitel	Laufzeit	Technologie^a^	Projektinformationen
AMICO – Personalisierte inklusive Assistenz durch adaptive Benutzerschnittstellen [16]	2024–2026	KI-gestützte Assistenzplattform: adaptive, behinderungsspezifische, multimediale und interaktive Benutzerschnittstellen und Anleitungen	*Zielgruppe:* Menschen mit sensorischen oder kognitiven Beeinträchtigungen *Einsatzbereich:* Produktion *Setting:* Werkstätten für behinderte Menschen (WfbM), Inklusionsunternehmen und andere Inklusionsorganisationen *Projektziele:* menschzentrierte Technologieentwicklung mit Fokus auf Partizipation, Adaption, teilautomatisierter Integration und Innovation
AWIEW – Arbeiten – wie ich es will [17]!	2021–2026	KI-gestütztes Bedarfsanalyse-Tool	*Zielgruppe:* Menschen mit Lernschwierigkeiten oder psychischen Beeinträchtigungen *Einsatzbereich:* Berufsorientierung *Setting:* WfbM *Projektziele:* Stärkung von Potenzialerkennung, Selbstbestimmung und Empowerment Unterstützung bei der beruflichen Orientierung Erleichterung der Arbeit von Fachkräften durch strukturierte Prozesse Erfassung von Wünschen, Stärken und Unterstützungsbedarfen durch Nutzende
AutARK – Automatische Adaption reizüberflutender Kontexte [18]	2023–2026	KI-gestützte Assistenzsysteme: Active-Noise-Cancellation-Verfahren Tragbare Assistenzgeräte	*Zielgruppe:* Menschen mit Autismus *Einsatzbereich:* Kommunikation, Erledigung von Aufgaben *Setting:* allgemeiner Arbeitsmarkt *Projektziele:* Verbesserung der Erwerbsfähigkeit und -tätigkeit durch: Reizminderung Unterstützung verbaler und textueller Kommunikation Unterstützung im Aufgaben- und Zeitmanagement Sensibilisierung der Interessengruppen (Stakeholder) für Bedarfe der Zielgruppe
CHAT-KI – Bessere Chancen für Menschen mit Behinderungen am Arbeitsmarkt durch KI-gestützte Informationen zu Unterstützungsleistungen [19]	2025–2026	KI-gestützter Chatbot: Audio-Ausgabemöglichkeiten	*Zielgruppe:* Menschen mit geistigen oder psychischen Beeinträchtigungen *Einsatzbereich:* nicht spezifiziert *Setting:* WfbM, allgemeiner Arbeitsmarkt *Projektziele:* Verbesserung des Zugangs zum allgemeinen Arbeitsmarkt für Menschen mit Behinderungen Verbesserung der Arbeitsbedingungen für Beschäftigte mit Behinderungen durch gezielte Informationsvermittlung und Assistenz Abbau des Fach- und Arbeitskräftemangels
EnAIble – Gestaltung eines inklusiven Arbeitsplatzes mit KI-Assistenz zur Qualitätskontrolle [20]	2025	KI-Assistenz zur Datenkontrolle basierend auf Computer-Vision	*Zielgruppe:* nicht spezifiziert *Einsatzbereich:* Montage *Setting:* WfbM *Projektziele:* Optimierung der Qualitätskontrolle Ermöglichung der stärkeren Übernahme von Verantwortung Förderung des kompetenten Umgangs mit KI-gestützter Software Schaffung eines neuen Arbeitsplatzes zur Befähigung mehrerer Beschäftigten für die Aufgabenausführung
Green ASC VET – Metautism [21]	2023–2026	KI-gestützte erweiterte Realitätstechnologien (Extended-Reality-(XR-)Tools) für das Kommunikationstraining^b^	*Zielgruppe:* Menschen mit Autismus *Einsatzbereich*: Berufsorientierung und allgemeine Arbeitskompetenzen *Setting:* berufliche Bildung *Projektziele:* Entwicklung von Berufsberatungs- und Qualifizierungsprogrammen für Menschen mit Autismus Weiterbildungsprogramme für Lehr- und Ausbildungskräfte Einsatz von XR-Technologien zur Selbstbestimmung Sensibilisierung von Arbeitgebenden für die Beschäftigung von Menschen mit Autismus
IIDEA – Inklusion und Integration durch Cobots auf dem ersten Arbeitsmarkt [22]	2025–2028	Cobot mit KI-gestützter Sprach- und Bilderkennung	*Zielgruppe:* nicht spezifiziert *Einsatzbereich:* Fertigung und Montage *Setting:* allgemeiner Arbeitsmarkt *Projektziele:* Etablierung kollaborativer Technologien in Unternehmen Förderung der Selbstbestimmung Stärkung der beruflichen Inklusion
InkluBot – Inklusive Sprachsteuerung für Roboter im Zeitalter von Large Language Models (LLMs; [23])	2026–2028	KI-gestützte Sprachsteuerung für Robotik auf Basis von großen Sprachmodellen (LLMs)	*Zielgruppe:* Menschen mit Sprachbeeinträchtigungen *Einsatzbereich:* Robotersteuerung *Setting:* WfbM *Projektziele:* Unterstützung der Interaktion mit Robotern Unterstützung der Problemlösung, Kommunikation und des Feedbacks Weiterentwicklung der KI durch spezifische Trainingsdatensätze von WfbM
KI.ASSIST – Assistenzdienste und Künstliche Intelligenz für Menschen mit Schwerbehinderung in der beruflichen Rehabilitation [24]	2019–2022	KI-gestützte Assistenzsysteme	*Zielgruppe:* nicht spezifiziert *Einsatzbereich:* nicht spezifiziert *Setting:* Berufsbildungswerk (BBW), Berufsförderungswerk (BFW), WfbM, allgemeiner Arbeitsmarkt *Projektziele:* Untersuchung der Potenziale von KI-Tools zur Unterstützung von Menschen mit Behinderungen bei der beruflichen Teilhabe Erprobung der Tools mit verschiedenen Zielgruppen in Lern- und Experimentierräumen Monitoring und Validierung bestehender KI-Tools
KI.inklusiv – KI-gestützte Assistenz zur Förderung dezentraler Inklusion [25]	2022–2026	Assistenzsystem mit KI-gestütztem Resilienzmanagementansatz	*Zielgruppe:* nicht spezifiziert *Einsatzbereich:* nicht spezifiziert *Setting:* WfbM *Projektziele:* Unterstützung der Beschäftigten beim selbstständigen Erlernen und Durchführen komplexer, variantenreicher Arbeitsprozesse Frühzeitige Antizipation von Unterstützungsbedarfen von Menschen mit Behinderungen in WfbM
KI-KOMPASS Inklusiv. – Kompetenzzentrum für KI-gestützte Assistenztechnologien und Inklusion in der Arbeitswelt [26]	2022–2027	KI-gestützte Assistenztechnologien	*Zielgruppe:* nicht spezifiziert *Einsatzbereich:* nicht spezifiziert *Setting:* nicht spezifiziert *Projektziele:* Bereitstellung einer Datenbank zu KI-gestützten Assistenztechnologien Bereitstellung von Schulungen und Beratung Entwicklung und Erprobung von KI-gestützten Assistenztechnologien
KomIn2Assist [27]	2023–2025	Kontextbewusstes Assistenzsystem: Spielerisch angereicherte Arbeitsprozesse („Gamification“) Textbasierte Interaktion (über Agenten mit Conversational User Interfaces) KI-gestützte Unterstützung zur Verbesserung von Einarbeitungsprozessen	*Zielgruppe:* nicht spezifiziert *Einsatzbereich:* (Klein‑)Montage *Setting:* WfbM und Inklusionsunternehmen *Projektziele:* Bereitstellung von Wissen und Coaching zur Stärkung von Kompetenz, Autonomie und Selbstvertrauen von Menschen mit Beeinträchtigungen
MIKE – Mehr Inklusion durch smarte KI auf Endgeräten [28]	2024–2026	Edge-KI-Systeme (Bereitstellung von KI auf lokalen Endgeräten) für spezifische Bedarfe von Menschen mit Behinderungen	*Zielgruppe:* nicht spezifiziert *Einsatzbereich:* nicht spezifiziert *Setting:* nicht spezifiziert *Projektziele:* Unterstützung von Autonomie und beruflicher Teilhabe auf dem allgemeinen Arbeitsmarkt
STARK-LS – Stärkung der Teilhabe auf dem ersten Arbeitsmarkt durch KI-generierte Leichte Sprache [29]	2025–2029	KI-gestützte Übersetzungssoftware für leichte Sprache	*Zielgruppe:* v. a. Menschen mit kognitiven Beeinträchtigungen *Einsatzbereich:* nicht spezifiziert *Setting:* Übergang in den allgemeinen Arbeitsmarkt *Projektziele:* Unterstützung von Arbeits- und Teamprozessen Kommunikation durch verständliche Arbeitsanweisungen und Schulungsmaterialien
TOP.Kl – Inklusive berufliche Prüfungen ohne Sprachbarrieren durch Textoptimierung mit Hilfe von Künstlicher Intelligenz [30]	2023–2026	KI-gestütztes Werkzeug zur Erstellung von Prüfungsfragen in einfacher Sprache für Auszubildende	*Zielgruppe:* v. a. Menschen mit Schwierigkeiten mit der Schriftsprache *Einsatzbereich:* Prüfungen *Setting:* berufliche Ausbildung *Projektziele:* Erleichterung des Textverständnisses bei gleichbleibendem Inhalt und Schwierigkeitsniveau Fokus auf Fach- und Anwendungswissen statt auf Textverständniskompetenz der Prüflinge
VisualGPT [31]	2025–2029	KI-gestützte multimodale Anwendung zur automatischen Übersetzung schriftsprachlicher Inhalte in visuelle Lernmaterialien und Deutsche Gebärdensprache	*Zielgruppe:* gehörlose Menschen *Einsatzbereich:* Lernen *Setting:* berufliche Qualifizierung *Projektziele:* Unterstützung durch barrierefreie Lernumgebung und Schulungen zur beruflichen Entwicklung

^a^ Angaben zu den Technologien entsprechend den Projekt-Webseiten

^b^ Gemäß mündlicher Aussage der Projektverantwortlichen ist das Tool KI-gestützt

**Tab. 2:** Eingeschlossene Projektberichte und wissenschaftliche Veröffentlichungen zu KI-Technologien im Arbeitskontext für Menschen mit Behinderungen im Zeitraum 2020–2025 in Deutschland

Autor:innen, Jahr	Studiendesign/Methode	Details zur Studie
Adolph et al. [32]	Exploratives Design: Expert:innenworkshops	*Technologie:* diverse KI-gestützte Tools (u. a. ChatGPT, JAWS, Tensor-Flow, Datenbrillen, Speech-to-text, AVA) *Anwendung: *z. B. E‑Mail-Kommunikation, Schreibunterstützung, Video-Konferenzen, Bilderkennung, Anleitungen, Montage, Programmierung, Kundenberatung, Recherchen, Bewerbungen *Zielgruppe:* Menschen mit Lernbeeinträchtigungen, Blindheit, Gehörlosigkeit *Setting:* nicht spezifiziert
Gerlmaier et al. [33]	Mixed-Methods: Erprobung und Evaluation eines Workshopprogramms	*Technologie:* Einführung eines Cobots mit KI-gestützter Bilderkennung *Anwendung:* Verklebungsarbeiten *Zielgruppe:* nicht spezifiziert *Setting:* WfbM
Gollasch et al. [34]	Literaturrecherche, Erprobung, Entwicklung, Evaluation	*Technologie:* auf Natural Language Processing (NLP) und Large Language Models (LLMs) gestützte KI-Tools *Anwendung:* E‑Mail-Korrespondenz (Strukturierung und automatisierte Extraktion von Aufgaben und Fristen) *Zielgruppe:* Menschen mit Autismus *Setting:* nicht spezifiziert
Hamideh Kerdar et al. [35]	Mixed Methods (mehrphasiges Design): Vorstudie, Entwicklung, Implementierung	*Technologie:* KI-gestützte Low-code/No-code-(LCNC-)Plattformen zur Entwicklung von Chatbots *Anwendung: *Entwicklung von Chatbots zu verschiedenen Themen: Urlaubsanträge, digitaler Kummerkasten, Online-Kochbuch, Krankheitsmeldung *Zielgruppe:* Menschen mit kognitiven Beeinträchtigungen, Lernschwierigkeiten oder körperlichen und entwicklungsbedingten Behinderungen *Setting:* WfbM
KI.ASSIST: Beudt et al. [36]	Literaturrecherche zu KI-gestützten Assistenzsystemen für Menschen mit Behinderungen und Experteninterviews	*Technologie:* KI-gestützte Anwendungen und Assistenztechnologien *Anwendung:* nicht spezifiziert *Zielgruppe:* nicht spezifiziert *Setting:* nicht spezifiziert
KI.ASSIST: Lippa und Stock [37]	Online-Befragung von Menschen mit Behinderungen zum Einsatz zweier ausgewählter KI-gestützter Assistenztechnologien	1. *Technologie:* OrCam MyEye 2 *Anwendung:* Sprachausgabe von Texten sowie Gesichts- und Produkterkennung *Zielgruppe:* Menschen mit Sehbehinderung oder Blindheit *Setting:* WfbM, BFW, BBW 2. *Technologie:* ADAMAAS – Adaptive and Mobile Action Assistance in Daily Living Activities (Datenbrille zur Einblendung von Informationen im Sichtfeld der Nutzenden) *Anwendung:* nicht spezifiziert *Zielgruppe:* Menschen mit kognitiven Beeinträchtigungen oder psychischen Erkrankungen *Setting:* WfbM, BFW, BBW
KI.ASSIST: Thieke-Beneke et al. [38] und Beudt et al. [39]	Design-Thinking zur Konzeption, Feldstudien zur Erprobung KI-gestützter Assistenztechnologien	Erprobungen: 1. *Technologie:* AirCrumb – App zu Tagesstrukturierung und Microlearning (KI-Komponenten: unter anderem Bild‑, Objekt- und Spracherkennung, Emotionserkennung, Anpassung an das Nutzungsverhalten) *Anwendung:* u. a. Tagesstrukturierung, Lernen, Aufgabenerledigung *Zielgruppe:* Menschen mit psychischen Beeinträchtigungen *Setting:* BBW 2. *Technologie:* EmpaT – Interaktive 3D-Trainingsumgebung für Bewerbungsgespräche (KI-Komponenten: trainiertes KI-Modell zur Interpretation von Emotionen über die Erkennung von Gestik, Mimik und Sprache) *Anwendung:* Training von Bewerbungsgesprächen mit virtuellen Avataren zur Verbesserung der Kommunikationskompetenz *Zielgruppe:* Menschen mit Kommunikationsschwierigkeiten *Setting:* BBW 3*.* *Technologie:* OPTAPEB – VR-Trainingsumgebung zur Emotionsbewältigung (KI-Komponenten: Emotionserkennung und -analyse mit Machine Learning und virtuelle Agenten) *Anwendung:* Stress- und Symptomreduktion bei sozialen Ängsten *Zielgruppe: *Menschen mit psychischen Erkrankungen (z. B. Angsterkrankungen, Depression) *Setting: *BBW 4. *Technologie:* ASSISTALL – Audio-Chatbot zur räumlichen Orientierung (KI-Komponenten: Audio-Chatbot, individuell trainiertes KI-Modell zur Dialogführung) *Anwendung:* Orientierung und Navigation in Gebäuden und auf Geländen *Zielgruppe: *Menschen mit Sehbeeinträchtigungen *Setting:* BFW 5. *Technologie:* EmmA – stationäres Biofeedback-Training mit digitalem Avatar (KI-Komponenten: trainiertes KI-Modell zur Interpretation von Herzratenvariabilität und Atemfrequenz der Nutzenden) *Anwendung:* Stress- und Selbstmanagement, Emotionsregulierung *Zielgruppe:* Menschen mit psychischen Erkrankungen (z. B. Burnout, Depression, Trauma) *Setting:* BFW, WfbM 6. *Technologie:* TeamViewer Frontline – Datenbrille mit Schritt-für-Schritt-Anleitungen per Augmented-Reality (AR) und Sprachbefehlen (KI-Komponenten: trainierbare Modelle zur Sprach- und Bilderkennung) *Anwendung:* Unterstützung bei Arbeitsprozessen, z. B. Montagearbeiten *Zielgruppe:* Menschen mit psychischen, kognitiven oder körperlichen Beeinträchtigungen, Menschen mit Lernschwierigkeiten *Setting:* BFW, WfbM 7. *Technologie:* INCLUSIFY – AR-App (KI-Komponenten: Bild‑, Objekt- und Spracherkennung) *Anwendung:* eigenständige Ausführung von Tätigkeiten, Lernen (in den Bereichen Arbeitssicherheit, Hygiene, hauswirtschaftliche Tätigkeiten oder Montage- und Konfektionierungsarbeiten) *Zielgruppe:* nicht spezifiziert *Setting:* WfbM

#### Projekte

Bei den Recherchen wurden 16 Projekte identifiziert, die sich im Zeitraum 2020 bis 2025 mit dem Thema KI-Technologien und berufliche Teilhabe befasst haben (Tab. 1). Etwa die Hälfte der Projekte fokussierte keine spezifische Behinderungsart. Bezogen auf das Setting waren weniger als die Hälfte der Projekte auf dem allgemeinen Arbeitsmarkt und 4 Projekte im Bereich der beruflichen Bildung und Qualifizierung von Menschen mit Behinderungen verortet.

Die Projektziele fokussierten zum einen auf die Förderung von Selbstbestimmung, Empowerment und Autonomie von Menschen mit Behinderungen, insbesondere durch die Stärkung individueller Handlungsspielräume. Außerdem wurde die Verbesserung der beruflichen Teilhabe sowie des Zugangs zum allgemeinen Arbeitsmarkt adressiert. In mehreren Projekten wurden darüber hinaus die Unterstützung bei komplexen Arbeitsabläufen und die Entlastung im Arbeitsalltag durch KI-gestützte Assistenzsysteme thematisiert. Weitere Ziele betrafen den Bereich Bildung, Qualifizierung und digitale Kompetenzentwicklung sowie die Unterstützung von Kommunikation durch verbesserte Barrierefreiheit.

Viele Projekte verfolgten einen partizipativen Ansatz in der Forschung oder Produktentwicklung, beispielsweise indem sie Rückmeldungen der Zielgruppe einbezogen und Produkte im realen Arbeitsumfeld erprobten. Teilweise zielten die Endergebnisse auf den Wissenstransfer ab, etwa in Form von Handlungsempfehlungen für Arbeitgebende. Da auf den meisten Projektwebseiten weder End- noch Zwischenergebnisse verfügbar waren, konnten Forschungsergebnisse nicht weiter bewertet werden.

#### Publikationen

Aus der Literaturrecherche ergaben sich 8 Veröffentlichungen, die den Ein- und Ausschlusskriterien entsprachen (Tab. 2). Die Hälfte der Veröffentlichungen stammt aus dem Projekt KI.ASSIST. Die meisten Veröffentlichungen bezogen sich auf das Setting der Werkstätten für behinderte Menschen (WfbM) oder Einrichtungen der beruflichen Rehabilitation. In 2 Veröffentlichungen war das Setting nicht spezifiziert. Abgesehen von KI.ASSIST waren die Stichproben der beteiligten Menschen mit Behinderungen klein.

### Thematische Analyse

**Abb. 1:**
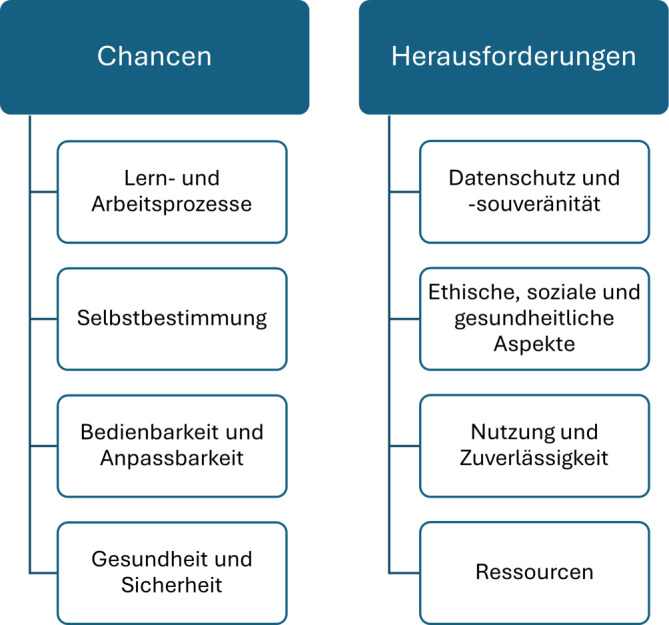
Chancen und Herausforderungen von KI-Technologien im Arbeitskontext für Menschen mit Behinderungen. Ergebnisse der thematischen Analyse der Publikationen

Die Studienergebnisse zeichnen ein vielschichtiges Bild von Chancen und Herausforderungen von KI für die berufliche Inklusion von Menschen mit Behinderungen. Entsprechend ihrer Thematik wurden die Ergebnisse den Hauptthemen Chancen und Herausforderungen zugeordnet und zusammengefasst. Abb. 1 zeigt eine Übersicht über die Haupt- und Subthemen der thematischen Analyse.

#### Chancen

##### Lern- und Arbeitsprozesse.

Die Ergebnisse der Publikationen zeigen, dass KI-gestützte Assistenztechnologien erweiterte Aufgaben- und Lernfelder eröffnen können [32, 36, 37]. Indem sie Leistungseinschränkungen ausgleichen und die Zugänglichkeit von Lern- und Arbeitsressourcen verbessern, können sie zum Abbau von Barrieren am Arbeitsplatz und in der Ausbildung beitragen [32–34, 36–39].

Ein wichtiger Anwendungsbereich liegt in der Unterstützung von Wahrnehmung und Kommunikation, die zugleich die Partizipation von Menschen mit Behinderungen an Besprechungen und Diskussionen fördern kann [32]. Beispiele sind Vorlesefunktionen bei Lese- oder Schreibschwierigkeiten, Transkriptionssoftware bei Hörbeeinträchtigungen oder Bilderkennung in Screenreadern bei Seheinschränkungen [32, 38]. Gollasch et al. [34] weisen zudem darauf hin, dass KI-Tools komplexe Inhalte und Handlungsanleitungen adaptiv strukturieren sowie sprachlich vereinfachen können, wodurch das Verständnis, die Aufgabenbearbeitung und die Effizienz des Aufgabenmanagements verbessert werden. Gleichzeitig können KI-Technologien wie Chatfunktionen die Kommunikation zwischen anwendenden Personen und Betreuungspersonal fördern [38].

Einige Studien führen an, dass KI-Technologien die realitätsnahe Gestaltung von Trainingsszenarien ermöglichen können. Avatare können etwa äußerliche Merkmale des beruflichen Umfelds abbilden und Informationen unmittelbar bereitstellen, wodurch die Authentizität des Lernfelds erhöht und der Lernprozess unterstützt werden kann [32, 38]. Für Lerninhalte, die den Einsatz beider Hände erfordern, oder für klar strukturierte Arbeitsabläufe wie in der Montage können KI-gestützte Augmented-Reality-Datenbrillen mit Schritt-für-Schritt-Anleitungen hilfreich sein [37, 38]. Gleichzeitig können digitale Anleitungssysteme mit KI-gestützter Bild‑, Objekt- und Spracherkennung Fehler reduzieren und die Arbeitsqualität sichern [38].

##### Selbstbestimmung.

Verbesserte Lern- und Arbeitsbedingungen durch KI-gestützte Assistenztechnologien (zum Beispiel Cobots) können Autonomie, Kompetenzentwicklung und soziale Eingebundenheit fördern und damit die Selbstbestimmung von Menschen mit Behinderungen stärken [33]. Digitale Anleitungs- und Rückmeldesysteme ermöglichen es Personen etwa, Aufgaben eigenständig zu bearbeiten und digitale Kompetenzen zu erwerben, ohne dauerhaft auf Unterstützung durch Betreuungspersonal oder begleitende Fachkräfte angewiesen zu sein [32, 33, 36, 38, 39].

Insbesondere das Lernen und Arbeiten im eigenen Tempo sowie der unabhängige Abruf von Lerninhalten können sich positiv auf die erlebte Selbstbestimmung auswirken [38]. Dabei sind eine effektive und selbstständige Nutzung KI-gestützter Assistenztechnologien sowie die eigenständige Gestaltung barrierearmer digitaler Werkzeuge vor allem dann möglich, wenn sie keine Programmierkenntnisse erfordern, leicht erlernbar und auf die spezifischen Bedürfnisse von Menschen mit Behinderungen zugeschnitten sind [35, 36, 38, 39]. Ein Beispiel hierfür liefern Hamideh Kerdar et al. [35] in ihrer Studie. Nach gezieltem Training konnten Menschen mit kognitiven Beeinträchtigungen und Lernschwierigkeiten eigenständig bedarfsorientierte Chatbots für ihre Arbeit entwickeln. Verwendet wurden KI-gestützte No-Code-Plattformen, die die Entwicklung von Apps und Webseiten durch vorgefertigte und modifizierbare Vorlagen und Funktionen ermöglichen [35].

##### Bedienbarkeit und Anpassbarkeit.

Eine wichtige Voraussetzung für die erfolgreiche Implementierung und Akzeptanz KI-gestützter Assistenztechnologien ist ihre intuitive Bedienbarkeit [33], für einige der untersuchten Technologien konnte diese in den einbezogenen Studien bestätigt werden [36, 38]. Zudem wird darauf hingewiesen, dass die Installation von KI-Software auf vertrauten, jederzeit zugänglichen Alltagstechnologien wie mobilen Endgeräten die Akzeptanz und Nutzungsbereitschaft von KI erhöhen kann – beispielsweise die Sprachsteuerung von Smartphones zur Informationsbereitstellung und zur räumlichen Orientierung für Menschen mit Sehbeeinträchtigungen [38].

Als besonderes Potenzial wird auch die individuelle Anpassung KI-gestützter Assistenztechnologien an die Bedarfe der Anwendenden hervorgehoben [32]. So können sich personalisierte KI-gestützte Assistenztechnologien dynamisch an individuelle Routinen und Anforderungen anpassen, direkte Rückmeldungen zu Aktivitäten geben, geeignete Zeitpunkte für Rückfragen identifizieren, an Termine erinnern oder Lernprozesse analysieren [32, 36, 38]. Durch diese bedarfsgerechte Anpassung und kontinuierliche Weiterentwicklung erscheint zudem ein langfristiger und nachhaltiger Einsatz solcher KI-gestützten Technologien im beruflichen Kontext möglich [36].

##### Gesundheit und Sicherheit.

Mehrere Studien heben das Potenzial von KI-gestützten Assistenztechnologien für Prävention und Arbeitsschutz hervor, indem sie körperliche und psychische Belastungen reduzieren und den Unfallschutz erhöhen [33, 34, 39]. So berichten Gerlmaier et al. [33], dass die Verletzungsgefahr und körperliche Zwangshaltungen an einem Heißklebearbeitsplatz durch einen KI-gestützten Cobot in Kombination mit einer humangerechten Gefährdungsbeurteilung gesenkt werden konnten. Zudem verringerte sich die Angst der Mitarbeitenden vor Fehlern und ihr Selbstwertgefühl wurde gestärkt. Des Weiteren können durch KI bereitgestellte Videoanleitungen zum Arbeitsschutz sicherheitsrelevante Inhalte auf verständliche Weise vermitteln [38].

Neben körperlichen Belastungen können auch kognitive Beanspruchungen gesenkt werden, indem KI-Tools Arbeitsinhalte vereinfachen oder Missverständnisse in der Kommunikation reduzieren [33, 34]. Ebenso lassen sich psychische Belastungen potenziell verringern, indem die selbstständige Stress- und Emotionsregulation unterstützt wird. Beispielsweise warnen KI-gestützte Biofeedbackgeräte anhand von Atem- und Herzfrequenzwerten frühzeitig vor hohen psychischen Belastungen und ermöglichen den Nutzenden, rechtzeitig geeignete Gegenmaßnahmen zu ergreifen [38, 39].

#### Herausforderungen

##### Datenschutz/-souveränität.

Die eingeschlossenen Publikationen verdeutlichen, dass die Nutzung von KI-Tools Datenschutzrisiken bergen kann [32, 38]. Beispiele hierfür sind die Umwandlung von gesprochener Sprache in Text, Visualisierungen von Arbeitsprozessen oder Zusammenfassungen von Online-Meetings, bei denen unternehmensinterne Inhalte oder Informationen Dritter verarbeitet werden. Zudem fehlen häufig inhaltlich ausreichende oder barrierefreie Informationen darüber, welche Daten von KI-Tools gesammelt, gespeichert und verarbeitet werden, und Nutzende können nicht aktiv über den Umfang der Datensammlung und -verarbeitung entscheiden [36]. Menschen mit Behinderungen selbst äußern Bedenken, dass ihre Privatsphäre nicht ausreichend geschützt und sensible Daten missbraucht werden könnten, indem sie Dritten zugänglich gemacht oder zur Überwachung genutzt werden [37].

##### Ethische, soziale und gesundheitliche Aspekte.

Eng verknüpft mit Datenschutzrisiken sind ethische Bedenken, da KI-Tools mitunter Daten sammeln, die Rückschlüsse auf persönliche und/oder gesundheitsbezogene Eigenschaften zulassen. Neben den erwähnten Sorgen der Menschen mit Behinderungen nennen die befragten Expert:innen im Projekt KI.ASSIST ebenfalls diese potenziellen Risiken und die damit einhergehende Diskriminierungsgefahr [36]. Zudem wird befürchtet, dass eine starke Fokussierung auf KI-gestützte Assistenztechnologien zum Verlust von Fähigkeiten oder Selbstständigkeit, zur Abhängigkeit von Technologien, zum Ersatz von Arbeitskräften oder zur Vernachlässigung menschlicher Interaktion, Unterstützung und Kommunikation durch KI führen könnte [32, 36, 37]. Soziale Spannungen könnten sich erhöhen [33] und die Teilhabe von Menschen mit Behinderungen am Arbeitsleben durch KI sogar negativ beeinflusst werden, wenn KI-Technologien beispielsweise primär zur Prozessautomatisierung eingesetzt werden [39].

Die Nutzung von KI-gestützten Assistenztechnologien kann außerdem gesundheitliche Auswirkungen auf die nutzenden Personen haben. So empfinden Anwendende mit Behinderungen beispielsweise körpernah genutzte Technologien wie Datenbrillen teils als belastend oder überfordernd [37, 38] oder sie erleben Stress bei der Interaktion mit KI-Tools wie Chatbots oder Avataren [32, 38]. Zudem äußern sie Bedenken, dass die sehr häufige Nutzung von KI-gestützten Technologien ihre körperlichen Einschränkungen verstärken könnte [37].

##### Nutzung und Zuverlässigkeit.

Barrierefreiheit ist für die Nutzung von KI-gestützten Assistenztechnologien durch Menschen mit Behinderungen entscheidend. Dabei ist zu unterscheiden, ob Systeme an sich technisch barrierefrei sind oder zusätzlich ohne Barrieren mit anderen Geräten (z. B. Hilfsmittel) oder Systemen verwendet werden können [32]. Eingeschränkt barrierefrei sind beispielsweise KI-Tools, die mit den Händen gehalten werden und somit von Menschen mit motorischen Beeinträchtigungen nur schwer nutzbar sind. Auch KI-Anwendungen mit integrierter Spracherkennung sind für Menschen mit Sprachbeeinträchtigungen oft nicht verwendbar [38]. Neben der mangelnden Barrierefreiheit können auch spezifische Anforderungen, zum Beispiel an digitale Kompetenzen zur Nutzung von Smartphones und Apps, Exklusionsrisiken erhöhen [38].

Die Zuverlässigkeit von KI-gestützten Assistenzsystemen ist ebenfalls kritisch zu bewerten [32]. Fehleranfällig sind etwa Tools zur Zusammenfassung oder Umwandlung von Informationen, da wichtige Inhalte verloren gehen oder fehlinterpretiert werden können. So erhalten beispielsweise blinde Menschen bei Videokonferenzen oder bei der Bilderkennung durch KI-gestützte Tools mitunter fehlerhafte oder unvollständige Informationen. Auch die Unterstützung beim Verständnis von E‑Mails für Menschen mit Autismus birgt Fehlerrisiken, wenn sich die Nutzenden ausschließlich auf KI verlassen [34]. Thieke-Beneke et al. [38] weisen zudem auf Herausforderungen bei KI-Chatbots hin, insbesondere wenn Nutzende nicht über die Möglichkeiten des Chatbots aufgeklärt werden und dessen Antworten nicht zufriedenstellend sind.

##### Ressourcen.

Die Implementierung und die Nutzung von KI-gestützten Assistenztechnologien können personelle, strukturelle und finanzielle Ressourcen erfordern. Das strategische Vorgehen bei der KI-Nutzung und die Bereitstellung der erforderlichen Ressourcen für die Einführung und Erprobung komplexer KI-gestützter Assistenztechnologien können insbesondere für kleinere Einrichtungen und Unternehmen herausfordernd sein [38, 39]. Hoher Ressourceneinsatz entsteht beispielsweise durch Tools, die an individuelle Arbeitsschritte, diverse Workflows und an individuelle Bedarfe der Nutzenden angepasst werden müssen und einen hohen Programmier- und Vorbereitungsaufwand erfordern [38]. Zudem können Schulungen zur effizienten und zielgerichteten Nutzung von KI-Tools, die Einarbeitung sowie die Erstellung von Lernmaterialien personelle und zeitliche Ressourcen binden [38].

Darüber hinaus erfordert die Einführung von KI-Tools, zum Beispiel in einer Werkstatt für behinderte Menschen (WfbM), eine hohe Einsatzbereitschaft aller Beteiligten, Veränderungen in der Organisationskultur sowie technische Ressourcen [35]. Dazu gehört eine effiziente IT- und Internet-Verfügbarkeit, die eine zielführende und selbstständige Nutzung von KI-Tools durch Menschen mit Behinderungen ermöglicht. Daneben entstehen in der Regel Kosten, beispielsweise für Anschaffung, Einrichtung und Wartung, Lizenzen sowie notwendige programmiertechnische Nachbesserungen durch Herstellende [34, 38].

## Diskussion

Die in diesem Review dargestellten Ergebnisse zeigen, dass KI-gestützte Technologien vielversprechende Potenziale für die inklusionsförderliche Arbeitsgestaltung und Chancengleichheit bieten, gleichzeitig aber mit multidimensionalen Herausforderungen verbunden sind. KI-gestützte Technologien können systematisch Barrieren, insbesondere im Bereich der Information und Kommunikation, abbauen und die Teilhabechancen verbessern [32, 34, 38]. In diesem Zusammenhang ermöglichen KI-gestützte Lösungen aufgrund ihrer hohen Verarbeitungsgeschwindigkeit, der Analyse großer Datenmengen sowie ihrer Fähigkeiten zur Automatisierung und individuellen Anpassung ein höheres Maß an Barrierefreiheit als frühere Technologien [40]. Über ihre kompensatorische Funktion hinaus können KI-gestützte Assistenztechnologien zudem die Selbstbestimmung von Menschen mit Behinderungen als stärkenorientiertes Leitziel von Inklusion fördern [37–39]. Die selbstständige und individualisierbare Nutzung von KI-gestützten Assistenztechnologien [35, 39] ist eine wichtige Voraussetzung für erlebte Selbstbestimmung, die eng mit psychologischem Wohlbefinden und Lebensqualität verknüpft ist [41, 42]. Andererseits können berufliche Teilhabechancen verringert werden, wenn KI primär zur Prozessautomatisierung und als Effizienzinstrument eingesetzt wird [32, 39]. Dies betonen auch Feichtenbeiner et al. [43], wonach ein verstärkter KI-Einsatz mit dem Verlust menschlicher Tätigkeiten und Kompetenzen sowie mit neuen technischen Abhängigkeiten einhergehen könnte.

Hervorzuheben ist, dass im Rahmen der betrachteten Projekte Menschen mit Behinderungen vielfach in unterschiedliche Projektphasen einbezogen wurden oder eine entsprechende Einbindung geplant ist. Beispielsweise erfasste ein Teil der Projekte systematisch die Bedarfe dieser Personengruppe, etwa durch das Einholen von Rückmeldungen in Pilotstudien oder durch eine aktive Beteiligung in Co-Design-Prozessen. Um Verzerrungen sowie daraus resultierende Diskriminierung und Exklusion einzelner Personengruppen zu vermeiden, sollten die Trainingsdaten der KI die Diversität der Gesellschaft und die individuellen Bedarfe abbilden. In diesem Zusammenhang betonen Goggin et al. [44] die Voreingenommenheit von KI gegenüber Menschen mit Behinderungen, die unter anderem auf nicht inklusive Trainingsdaten zurückzuführen ist. Positiv anzumerken ist, dass einige Projekte versuchten, diesem Risiko entgegenzuwirken. Sie trainierten KI-Systeme beispielsweise mit Daten ihrer jeweiligen Zielgruppe (z. B. WfbM) und reflektierten ihre Weiterentwicklung entsprechend.

Das Review verdeutlicht, dass sich die meisten Veröffentlichungen und Projekte – abgesehen vom Projekt KI.ASSIST – auf die Entwicklung und das Training neuer, auf die spezifischen Bedürfnisse von Menschen mit Behinderungen zugeschnittener KI-gestützter Assistenztechnologien konzentrierten. Diese Technologien können die Gebrauchstauglichkeit erhöhen, Barrierefreiheit verbessern und die Akzeptanz bei den Nutzenden steigern. Gleichzeitig besteht empirisches Forschungspotenzial zu Mainstream-KI-Tools. Generative KI wird auch von Personen ohne technische Vorkenntnisse, einschließlich von Menschen mit Behinderungen etwa zur Unterstützung beim akademischen Schreiben, genutzt [45]. Dadurch etabliert sich generative KI zunehmend als niedrigschwellig nutzbare und barrierearme Technologie, trotz bestehender Widersprüche oder sogenannter Halluzinationen [46]. In Deutschland liegen jedoch kaum Forschungsarbeiten zur Nutzung solcher KI-gestützten Technologien durch Menschen mit Behinderungen im Arbeitskontext vor. Studien dazu sind jedoch wichtig, um neben den Potenzialen von KI für Menschen mit Behinderungen auch kritische Aspekte wie Datenschutz und Datensouveränität sowie ethische Fragestellungen zu berücksichtigen. Hinzu kommt, dass die digitalen Kompetenzen der Nutzenden entscheidend sind im Hinblick auf die sichere Nutzung von KI-Technologien und damit auch für den Schutz von personenbezogenen Daten.

Trotz des umfassenden Überblicks über den aktuellen Forschungsstand zu KI für die berufliche Teilhabe in Deutschland weist dieses Review einige Limitationen auf. Durch den Fokus auf Publikationen und Projekte in Deutschland können weitere Perspektiven, z. B. aus internationalen Studien, nicht erfasst sein. Da die Hälfte der Publikationen aus dem Projekt KI.ASSIST stammt, kann eine Verzerrung bei Auswertung und Interpretation der Ergebnisse nicht ausgeschlossen werden. Auch bei einer umfassenden Suchstrategie ist es möglich, dass einzelne Publikationen oder Projekte nicht identifiziert wurden, insbesondere wenn der Einsatz von KI nicht explizit in Beschreibungen und Schlüsselbegriffen genannt ist. Einigen Projekten fehlten Webseiten oder sie ließen sich nicht durch Datenbankrecherchen identifizieren.

### Wissenschaftliche Implikationen.

Die begrenzten wissenschaftlichen Veröffentlichungen in Deutschland weisen auf weiteren Forschungsbedarf zum untersuchten Thema hin. Für die zukünftige Forschung sind langfristig angelegte Studien mit größeren Stichproben empfehlenswert. Aufschlussreich wären empirische Untersuchungen zu den Auswirkungen von KI-Anwendungen, die Einflussfaktoren systematisch erforschen. Mögliche Schwerpunkte wären z. B. die Zusammenarbeit in Teams oder eine stärkere Berücksichtigung des allgemeinen Arbeitsmarkts, die in Studien und Projekten bislang unterrepräsentiert sind. Zukünftige Forschung sollte darüber hinaus einen multiperspektivischen und interdisziplinären Ansatz verfolgen. Dabei sollten neben der individuellen und technischen Ebene auch die organisationalen Rahmenbedingungen wie beispielsweise Datenschutzregelungen betrachtet werden.

Um ein umfassenderes und realitätsnahes Bild des Einsatzes von KI für Menschen mit Behinderungen zu gewinnen, sollten partizipative Ansätze in realen Arbeitsumgebungen verfolgt werden. Diese tragen dazu bei, den Transfer von Forschungsergebnissen in die Praxis anhand der Bedarfe und Anregungen der Zielgruppe sicherzustellen. Daher sollte die Zielgruppe aktiv und möglichst in alle Phasen der Studien einbezogen werden.

### Praktische Implikationen.

Bei der Nutzung von digitalen Technologien, einschließlich KI-gestützter Tools, ist Barrierefreiheit für Menschen mit Behinderungen entscheidend. Unternehmen sollten deren Belange bereits vor der Entscheidung für eine Technologie aktiv erheben und in die Entwicklung und Implementierung einfließen lassen. So sollte sichergestellt werden, dass beispielsweise Nutzende von Screenreadern KI-Tools problemlos anwenden können. Darüber hinaus bieten Plattformen wie REHADAT^3^ und der Technologie-Monitor des Projekts KI-Kompass Inklusiv^4^ unabhängige Übersichten zu KI-gestützten Assistenztechnologien und Projekten für beteiligte Akteure.

Angesichts der schnellen Entwicklung KI-gestützter Tools können Schulungen für deren Bedienung hilfreich sein. Zudem ist es wichtig zu vermitteln, wie sich KI-Technologien in Kombination mit anderen Unternehmens-Tools oder Hilfsmitteln nutzen lassen. Dabei sollte auch für die Herausforderungen sensibilisiert werden, die etwa durch den Einsatz generativer KI am Arbeitsplatz entstehen können.

## Fazit

Die Ergebnisse zeigen Möglichkeiten, wie KI-gestützte Assistenztechnologien eine inklusive Arbeitswelt für Menschen mit Behinderungen fördern können. Sie richten sich an Akteure aus Politik, Wirtschaft, Bildung, Forschung und Entwicklung sowie an Menschen mit Behinderungen. Dabei ist es jedoch notwendig, KI-gestützte Assistenztechnologien nicht ausschließlich als technische Hilfsmittel zu betrachten, sondern sie in ein umfassendes inklusives Gesamtkonzept einzubetten. Um die Potenziale der KI für eine inklusive Arbeitswelt auszuschöpfen und den Herausforderungen mit adäquaten Lösungen zu begegnen, braucht es zudem partizipative Entwicklungs- und Implementierungsprozesse, datenschutzrechtliche und ethische Leitlinien sowie gleichberechtigte Zugangsmöglichkeiten für alle Menschen.

## Data Availability

Die während der vorliegenden Studie erzeugten und/oder analysierten Datensätze sind auf begründete Anfrage bei der Korrespondenzperson erhältlich.

## Footnotes

1 www.daaap.net (zugegriffen: 28.01.2026).

2 www.ki-assist.de (zugegriffen: 30.01.2026).

3 www.rehadat.de (zugegriffen: 30.01.2026).

4 www.ki-kompass-inklusiv.de (zugegriffen: 30.01.2026).
